# Medical Evacuations out of U.S. Central and U.S. Africa Commands Among the Active and Reserve Components of the U.S. Armed Forces, 2024

**Published:** 2025-09-20

This report summarizes the nature, numbers, and trends of conditions for which military members were medically evacuated from the U.S. Central Command (CENTCOM) or Africa Central Command (AFRICOM) operations in 2024, with historical comparisons to the previous 4 years. During deployed military operations, initial medical care is provided by military medical personnel stationed within the operational theater, but some injuries and illnesses require medical care outside the theater of operation. In such cases, affected individuals may be transported to a permanent military medical facility, usually in Europe or the U.S., for definitive diagnosis or care. Because medical evacuations are resource-intensive, they are employed for serious medical conditions, some of which are directly related to participation in, or support of, military operations. Other medical conditions that are unrelated to operational activities but necessitate medical evacuation may be preventable.

With completion of the withdrawal of all U.S. military forces from Afghanistan on August 31, 2021, followed by the conclusion of the U.S. combat mission in Iraq on December 9, 2021, ^1 , 2^ U.S. military operations were substantially reduced in the CENTCOM area of responsibility (AOR). To sustain counterterrorism operation successes, force deployment continues in all AORs, in addition to assistance, advice, and accompaniment of selected partners' security forces. ^3^

This report only includes medical evacuations from CENTCOM and AFR-ICOM, without describing any medical evacuations from troop deployment to the U.S. European Command (EUCOM), U.S. Indo-Pacific Command (INDOPACOM), or U.S. Southern Command (SOUTH-COM). *MSMR* has historically reported medical evacuations from CENTCOM due to large numbers of service members deployed for named operations including Operation Iraqi Freedom, Operation Enduring Freedom, and Operation New Dawn. The AFRICOM AOR was added to this annual report in 2021 due counterterrorism force deployment. ^3^ Future reports may review medical evacuations from other AORs, as required by leadership interest or changing operational tempos.

## Methods

The surveillance population for this analysis includes all members of the active and reserve components of the U.S. Army, Navy, Air Force, Space Force, and Marine Corps deployed to the CENTCOM or AFRICOM AORs for any length of time from January 1, 2020 through December 31, 2024. Medical evacuations by the U.S. Transportation Command (TRANSCOM) from the CENTCOM or AFRICOM AORs to a medical treatment facility outside the operational theater were assessed from records maintained in the TRANSCOM Regulating and Command & Control Evacuation System (TRAC2ES). CENTCOM and AFRICOM evacuation data are presented separately.

Medical evacuations were classified by the cause and nature of the precipitating medical condition, based on information in relevant evacuation and medical encounter records. All medical evacuations were classified as battle injuries or non-battle injuries and illnesses, based on entries in the TRAC2ES evacuation record. Evacuations due to non-battle injuries and illnesses were further classified into 18 illness and injury categories based on International Classification of Diseases, 9th and 10th Revisions (ICD-9 and ICD-10, respectively) diagnostic codes reported in medical encounter records following evacuation.

**What are the new findings?**

Non-battle injuries constituted the most frequent diagnostic categories for service members medically evacuated in 2024 from U.S. Central Command (CENTCOM) and U.S. Africa Command (AFRICOM). Of the 714 CENTCOM service members and 171 AFRICOM service members evacuated for medical reasons in 2024, hospitalization was required for 228 (31.9%) and 42 (24.6%), respectively. Most service members evacuated from CENTCOM or AFRICOM were returned to full duty status after their post-evacuation hospitalizations or outpatient evaluations.

**What is the impact on readiness and force health protection?**

In 2024, evacuations for disease and non-battle injuries from U.S. CENTCOM and AFRICOM were similar to numbers observed in 2022 and 2023. Non-battle injuries and mental health disorders are the leading causes for medical evacuations and should remain the focus for future prevention efforts.

All records of hospitalizations and ambulatory visits at a permanent military medical facility in the U.S. or Europe within an interval of 5 days preceding to 10 days following the reported date of each medical evacuation were identified from Defense Medical Surveillance System (DMSS) data. The primary (i.e., first-listed) diagnosis for either hospitalization or earliest ambulatory visit after evacuation was used to classify the condition that necessitated the evacuation. If the first-listed diagnostic code specified an external cause of injury (ICD-9 ‘E’ code, ICD-10 ‘V’, ‘W’, ‘X’, or ‘Y’ codes) or an encounter for a condition other than a current illness or injury, the secondary diagnosis specifying illness or injury (ICD-9, 001–999; ICD-10, A00–T88, U07.1, U09.9) was used. If no secondary diagnosis was provided, or if the secondary diagnosis also was an external cause code, the first-listed diagnostic code of a subsequent encounter was used.

## Results

In 2024, there were 714 medical evacuations from the CENTCOM AOR and 171 from the AFRICOM AOR. These medical evacuations were required to be associated with at least 1 subsequent medical encounter at a permanent medical facility outside the operational theater, within the requisite inclusion timeframe **(Table 1)** . Non-battle injuries accounted for the most medical encounters after an evacuation from both CENTCOM (n=198, 27.7%) and AFRICOM (n=52, 30.4%) **(Table 1)** . Mental health disorders accounted for the second-most medical encounters following a CENTCOM evacuation (n=196, 27.5%).

**TABLE 1.** Numbers and Percentages of Medical Encounters Following Medical Evacuation ^a^ from Theater, by Area of Responsibility and Major ICD-10 Diagnostic Category, U.S. Armed Forces, 2024

	CENTCOM						AFRICOM					
	Total		Men		Women		Total		Men		Women	
Major Diagnostic Category (ICD-10 codes)	No.	%	No.	%	No.	%	No.	%	No.	%	No.	%
Non-battle injury, poisoning (ICD-10: S00–T88, DOD0101–DOD0105)	198	27.7	174	30.9	24	16.0	52	30.4	45	30.2	7	31.8
Mental disorders (ICD-10: F01–F99)	196	27.5	146	25.9	50	33.3	21	12.3	17	11.4	4	18.2
Musculoskeletal system (ICD-10: M00–M99)	77	10.8	65	11.5	12	8.0	20	11.7	19	12.8	1	4.5
Signs, symptoms, ill-defined conditions (ICD-10: R00–R99)	70	9.8	51	9.0	19	12.7	25	14.6	23	15.4	2	9.1
Digestive system (ICD-10: K00–K95)	40	5.6	34	6.0	6	4.0	14	8.2	11	7.4	3	13.6
Nervous system and sensory organ disorders (ICD-10: G00–G99, H00–H95)	29	4.1	21	3.7	8	5.3	13	7.6	10	6.7	3	13.6
Battle injuries (from TRAC2ES records)	20	2.8	16	2.8	4	2.7	0	0.0	0	0.0	0	0.0
Circulatory system (ICD-10: I00–I99)	18	2.5	16	2.8	2	1.3	3	1.8	3	2.0	0	0.0
Genitourinary system (ICD-10: N00–N99)	16	2.2	5	0.9	11	7.3	5	2.9	4	2.7	1	4.5
Respiratory system (ICD-10: J00–J99, U07.0)	12	1.7	11	2.0	1	0.7	1	0.6	1	0.7	0	0.0
Neoplasms (ICD-10: C00–D49)	9	1.3	7	1.2	2	1.3	3	1.8	3	2.0	0	0.0
Other (ICD-10: Z00–Z99, except pregnancy-related)	7	1.0	1	0.2	6	4.0	6	3.5	5	3.4	1	4.5
Endocrine, nutritional, metabolic diseases (ICD-10: E00–E89)	6	0.8	5	0.9	1	0.7	1	0.6	1	0.7	0	0.0
Skin and subcutaneous tissue diseases (ICD-10: L00–L99)	6	0.8	5	0.9	1	0.7	2	1.2	2	1.3	0	0.0
Infectious and parasitic diseases (ICD-10: A00–B99)	4	0.6	3	0.5	1	0.7	5	2.9	5	3.4	0	0.0
Congenital anomalies (ICD-10: Q00–Q99)	3	0.4	3	0.5	0	0.0	0	0.0	0	0.0	0	0.0
Pregnancy and childbirth (ICD-10: O00–O9A, relevant Z codes)	2	0.3	0	0.0	2	1.3	0	0.0	0	0.0	0	0.0
Hematological disorders (ICD-10: D50–D89)	1	0.1	1	0.2	0	0.0	0	0.0	0	0.0	0	0.0
COVID-19 (U07.1, U09.9)	0	0.0	0	0.0	0	0.0	0	0.0	0	0.0	0	0.0
Total	714	100	564	100	150	100	171	100	149	100	22	100

Abbreviations: CENTCOM, U.S. Central Command; AFRICOM, U.S. African Command; ICD, International Classification of Diseases, 10th Revision; No., number; TRAC2ES, U.S. Transportation Command (TRANSCOM) Regulating and Command & Control Evacuation System.

a Classified as Disease and non-Battle Injuries from ‘injury_type’ field in TRAC2ES

Annual CENTCOM medical evacuations attributable to battle injuries were highest in 2020 (n=59) and subsequently decreased in 2021 (n=7), 2022 (n=3), 2023 (n=14) and 2024 (n=20), following the conclusion of major combat operations (data not shown). Annual CENTCOM medical evacuations attributable to non-battle injuries also declined, from 1,134 to 694 during the 2020–2024 surveillance period **(Figure)** . Annual medical evacuations from AFRICOM attributable to battle injuries peaked at 4 in 2020, falling below this number in 2021 (n=1), 2022 (n=2), 2023 (n=1) and 2024 (n=0) (data not shown). Notably, the annual number of AFRICOM medical evacuations attributable to non-battle injuries and diseases remained much lower than CENTCOM during the 2020–2024 surveillance period **(Figure)** .

**FIGURE:**
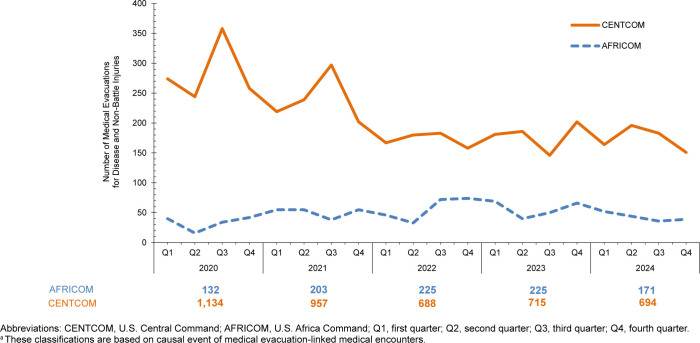
Numbers of Medical Evacuations of U.S. Service Members for Disease and Non-Battle Injuries, by Area of Responsibility and Quarter Year, 2020–2024 ^a^

### Demographic and military characteristics

The leading major diagnostic categories following evacuations from CENT-COM in 2024 were non-battle injuries for men (n=173, 30.7%) and mental disorders for women (n=49, 32.9%). In AFRICOM, the leading major diagnostic categories in 2024 were non-battle injuries for both men (n=45, 30.2%) and women (n=7, 31.8%) **(Table 1)** . Female CENTCOM service members had a higher proportion of medical evacuations for mental health disorders compared to male CENTCOM service members (32.9% and 25.8%, respectively) **(Table 1)** .

The largest numbers and proportions of evacuees from CENTCOM and AFRI-COM involved non-Hispanic White service members, those aged 20-24 years, members of the Army, and senior enlisted personnel. Most medical evacuations from CENT-COM (86.7%) and AFRICOM (85.4%) were assigned routine precedence **(Table 2)** .

**TABLE 2.** Demographic and Military Characteristics of Service Members Medically Evacuated from the U.S. Central and Africa Command Areas of Responsibility, U.S. Armed Forces, 2024

	CENTCOM		AFRICOM	
	No.	%	No.	%
Total	714	100	171	100
Sex				
Male	564	79.0	149	87.1
Female	150	21.0	22	12.9
Age group, y				
<20	12	1.7	6	3.5
20–24	202	28.3	44	25.7
25–29	170	23.8	30	17.5
30–34	124	17.4	32	18.7
35–39	107	15.0	31	18.1
40–44	56	7.8	10	5.8
45+	43	6.0	18	10.5
Race and ethnicity				
White, non-Hispanic	353	49.4	101	59.1
Black, non-Hispanic	168	23.5	31	18.1
Hispanic	111	15.5	30	17.5
Other / unknown	82	11.5	9	5.3
Service				
Army	465	65.1	93	54.4
Navy	101	14.1	28	16.4
Air Force	138	19.3	43	25.1
Marine Corps	10	1.4	7	4.1
Component				
Active	347	48.6	57	33.3
Reserve, Guard	367	51.4	114	66.7
Rank, grade				
Junior enlisted (E1–E4)	279	39.1	57	33.3
Senior enlisted (E5–E9)	341	47.8	97	56.7
Junior officer (O1–O3; W1–W3)	70	9.8	6	3.5
Senior officer (O4–O10; W4–W5)	24	3.4	11	6.4
Military occupation				
Combat-specific ^a^	103	14.4	42	24.6
Motor transport	42	5.9	2	1.2
Pilot, air crew	16	2.2	1
Repair, engineering	197	27.6	40	23.4
Communications, intelligence	164	23.0	41	24.0
Health care	61	8.5	6	3.5
Other / unknown	131	18.3	39	22.8
Marital status				
Married	344	48.2	87	50.9
Single, never married	319	44.7	68	39.8
Other / unknown	51	7.1	16	9.4
Education				
High school or less	452	63.3	96	56.1
Some college	76	10.6	22	12.9
College	153	21.4	41	24.0
Other / unknown	33	4.6	12	7.0
Precedence ^b^				
Routine	619	86.7	146	85.4
Priority	84	11.8	20	11.7
Urgent	11	1.5	5	2.9
Transportation mode				
Military	483	67.6	38	22.2
Commercial	26	3.6	5	2.9
Other / unknown	205	28.7	128	74.9

Abbreviations: CENTCOM, U.S. Central Command; AFRICOM, U.S. Africa Command; No., number; y, years.

a Infantry / artillery / combat engineering / armor.

b Data field within U.S. Transportation Command (TRANSCOM) Regulating and Command & Control Evacuation System (TRAC2ES).

### Most frequent specific diagnoses

Among men and women in both AORs, the leading 3-digit ICD-10 code for mental health disorders (F43) indicated reaction to severe stress and adjustment disorders **(Table 3)** . This ICD-10 code represented over two-thirds of the mental disorder diagnoses among men in CENTCOM and women in both AORs (data not shown). In CENTCOM, evacuations for other joint disorders and wrist/hand fractures were the second- and third-most common 3-digit ICD-10 codes for men **(Table 3)** .

**TABLE 3.** Most Frequent Three-Digit ICD-10 Diagnoses Associated with Medical Evacuations, by Area of Responsibility and Sex, U.S. Armed Forces, 2023

CENTCOM					
Males			Females		
3-Digit ICD-10 Code	ICD-10 Code Description	No.	3-Digit ICD-10 Code	ICD-10 Code Description	No.
F43	Reaction to severe stress, adjustment disorders	109	F43	Reaction to severe stress, adjustment disorders	35
M25	Other joint disorder, not elsewhere classified	26	M25	Other joint disorder, not elsewhere classified	8
S62	Fracture, wrist and hand	23	F32	Depressive episode	6
S83	Dislocation and sprain of joints and ligaments of knee	17	R10	Abdominal and pelvic pain	5
M54	Dorsalgia	13	S83	Dislocation and sprain of joints and ligaments of knee	4
**AFRICOM**					
**Males**			**Females**		
**3-Digit ICD-10 Code**	**ICD-10 Code Description**	**No.**	**3-Digit ICD-10 Code**	**ICD-10 Code Description**	**No.**
F43	Reaction to severe stress, adjustment disorders	8	F43	Reaction to severe stress, adjustment disorders	4
M54	Dorsalgia	7	G45	Transient cerebral ischemic attacks and related syndromes	1
M25	Other joint disorder, not elsewhere classified	6	H00	Hordeolum and chalazion	1
S62	Fracture, wrist and hand	6	H52	Disorders of refraction and accommodation	1
Z02	Encounter for administrative examination	5	K35	Acute appendicitis	1

Abbreviations: ICD, International Classification of Diseases, 10th Revision; CENTCOM, Central Command; AFRICOM, Africa Command; No., number.

### Disposition

Hospitalization was required for 228 (31.7%) of CENTCOM (n=714) and 42 (24.6%) of AFRICOM (n=171) medical evacuees in 2024 (data not shown).

## Discussion

In 2024, only 20 (2.8%) medical evacuations from CENTCOM and none (0) from AFRICOM were associated with battle injuries in TRAC2ES records. Evacuations for disease and non-battle injuries from CENTCOM and AFRICOM in 2024 remained similar to numbers observed in 2022 and 2023. These trends reflect the continued counterterrorism force deployment throughout CENTCOM and AFRICOM AORs. ^3^

The leading diagnoses of AFRICOM non-battle injuries were not clustered around any specific ICD-10 code but were distributed among diagnoses such as muscle and tendon injuries and fractures. This heterogeneity of injury type may be due to the large proportion due to occupational hazards in the deployed environment. Classification by cause of injury, rather than affected body system, may be more appropriate for this population; the ICD chapter for external causes of morbidity codes is intended for secondary coding purposes and is not mandatory, however. Consequently, completeness and specificity of these external cause codes for injury-related diagnoses may vary according to coding practices. ^4^

The leading diagnoses of CENTCOM non-battle injuries were also heterogenous and included unclassified joint disorders, fractures, dislocation and sprains, and tendon injuries. The proportion of CENTCOM medical evacuations attributed to mental health disorders in 2023 (27.5%, n=199) and 2024 (27.5%, n=196) represents a sustained decline after increasing proportional trends reported in 2020 (27.1%, n=323), 2021 (33.3%, n=321), and 2022 (38.6%, n=267). ^5 - 8^ The proportions of medical evacuations due to mental health disorders are considerably higher than the proportion (11.6%, n=5,892) described by a *MSMR* report that examined evacuations from Iraq during a 9-year period from 2003 through 2011. ^9^

Several important limitations should be considered when interpreting these results. Demographic data for the deployed population, i.e., person-time for individuals eligible for medical evacuation, are not readily available. The lack of deployed individual person-time precludes calculation of stratified and overall rates for medical evacuations.

Most causes of medical evacuations were estimated for this report from primary (i.e., first-listed) diagnoses in DMSS recorded during hospitalizations or initial outpatient encounters following evacuation. Diagnoses recorded in theater through the Theater Medical Data Store (TMDS) are not reflected in this analysis. In some cases, clinical evaluations at fixed medical treatment facilities following medical evacuation may have eliminated serious conditions that were clinically suspected while in theater, resulting in possible misclassification errors.

Battle injuries rely on proper classification in the TRAC2ES system. Misclassification errors may occur, and given the small number of battle injuries, any misclassification will have a disproportionate effect.
